# Physical Parameters of Blood as a Non - Newtonian Fluid

**Published:** 2008-12

**Authors:** H. E. Abdel Baieth

**Affiliations:** *Faculty of Science in Dammam, King Faisal University, Physics department, Saudi Arabian Kingdom*

**Keywords:** Viscosity of blood, electromagnetic field, permittivity of RBCs, molecular radius of RBCs, flow of blood, conductivity

## Abstract

Do increasing doses of electromagnetic fields (EMFs) increase the hazards effect on blood? Studies on the blood of rats provide guidance for the assessment of occupational and public health significance of exposure to EMFs. Here, apparent additive viscosity of animal blood after exposing to EMFs (3,5 and 10 gauss) is examined. The results indicate that hematocrite (HCT) increased as EMF increases while the viscocity decreased with the increase of EMF. Red blood cell permeability, deformability and the electrical properties of hemoglobin (conductivity and relaxation time) were also examined. The results show that EMF produces pronounced changes in the molecular structure of hemoglobin and induced force acting on the charged particle of charge q which may activate Rouleau formation of red blood cells (RBCs).

## INTRODUCTION

Many researchers have investigated studied the influence of electromagnetic fields (EMF) on nucleic acid and proteins. Walter *et al* (24), MacGinitics *et al* (15) and Leberge (13) investigated the effects of EMF on the biological properties of membrane proteins. Fawzia (5) investigated the effects of such field (50 Hz) on the bone marrow of the mice, showing significant increases in the frequency of the chromosomal aberrations (CA) and micronuclei polychromatic erythrocyte (MNPC) with increasing the time of exposure. Tenford (20) noted that an extremely low frequency (ELF) induces electrical potentials in the aqueous medium surrounding living cells. It is believed that the current induced by ELF field produces electrochemical alterations in cell membrane surfaces.

Early on, blood was treated as a Newtonian fluid (14). However Thurston (21) reported that viscoelasticity is known to be a basic rheological property of blood. The viscoelastic properties which make human blood non- Newtonian depend on the elastic behavior of red blood cells.

Recently interest in problems of non-newtonian fluid have grown and many mathematical models for describing the rheological behavior of blood have been extensively developed (1, 16, 17, 23). Haik *et al* (10) studied the effect of high static field (3, 5 and 10 mTesla) on the apparent viscosity of human blood, finding an increase in blood viscosity under the influence of magnetic fields. This behavior was related to magnetic torque, which induces the cells to orient. Therefore, in an effort to obtain more information and understanding about the effect of EMFs on the biological systems, we examined the dependence of blood viscosity on the parameters emerging in the constitutive model and applied magnetic field.

## MATHEMATICAL MODEL

### Viscosity of blood

Recently interest in problems regarding non-newtonian fluids has grown considerably, particularly due to their medical applications. However there is not a single governing constitutive equation describing all the properties of the non- Newtonian fluids (the blood at present).

Due to this, many constitutive equations for non–Newtonian fluids have been proposed.

The flow of an electrically conducting incompressible fluid is governed Maxwell's equations (12):

(a) $$\rho \left[\partial V/\partial t+\left(V\cdot \nabla \right)V\right]=\nabla \cdot T+J\times \beta$$

(b) $$\nabla \cdot V=0,\nabla \cdot \beta =0,\nabla \times \beta =\mu_{m}J$$

(c) ∇×E=−∂β/∂t J=σE

where V is the velocity vector field, ρ the density, J the current density, β the total magnetic field, μ_m_ the magnetic permeability, E the electric field and σ the electric conductivity. The electric field is assumed to be zero, so that electromagnetic body force in equation (a) becomes:

(d) $$J\times \beta =-\sigma \beta_{o}^{2}$$

Mathematical models of blood flow constitute an alternative and useful tool for supporting experiments and detect minor parameters which are not obvious by measurements. The viscosity of blood is considered as a decreasing function of shear rate γ°. General regression analysis of experimental data suggests the use of some complicated transcendental functions for η (γ°) (18). The apparent viscosity has two distinct values η_o_ and η_∞_ as γ°→0 and γ°→∞ respectively. The analytical function which offers the best fit for experimental data with a large range of flows has been found to be:

(e) $$\eta \left(\gamma^{o}\right)=\eta_{\infty }+\left(\eta_{o}-\eta_{\infty }\right)\left\{\left[1+\log\left(1+{\Lambda \gamma }^{o}\right)\right]/\left(1+{\Lambda \gamma }^{o}\right)\right\}$$

where Λ is a material constant with the dimension of time. Experimental results show that blood is not a purely viscose fluid, but possesses significant viscoelastic properties (21).

The viscosity of blood is a determinant shear stress where the shear stress is the energy transferred to the vessel wall due to interaction with a fluid in motion.

(f) $$T=\eta \times \gamma^{o}$$

where T is the shear stress, η is the viscosity and γ° is the shear rate γ° = ∂v (r) / ∂r where ∂v (r) / ∂r is related to the flow velocity. While the exposure to EMF induced a force (F) acting on the charged particles of charge q, equation (f) should be as follow:

(g) $$\eta =\left(\tau +F\right)/\gamma^{o}$$

where F = q (E + v × β) is the Lorentz force.

### Electrical properties of blood

Hemoglobin (Hb) solution was prepared according to the method of Trivelli (22). Dielectric measurements were made on the collected hemoglobin from the different groups of rats within frequency range from 50 Hz to 200 kHz using LCZ meter type Chen Hwa 1061 manufactured by Taiwan IEEE-488 interface, with a conductivity cell type PW 950/60 manufactured by Philips, Holland. The cell has two parallel squared black electrodes of 0.8 cm side each and area 0.64 cm^2^ (A), and 1 cm separation distance (d).

Dielectric measurements were carried out at 4° ± 0.1°C. The measured values of capacitance (c) and resistance (R) were used to calculate the real (ε′) and imaginary (ε″) parts of the complex permittivity and the conductivity σ.

The dielectric constant (ε′) and ac conductivity (σ) for samples were calculated at each frequency from the measured value of the capacitance (C) through the equation:

(h) $$\varepsilon^{/}=c/{k\varepsilon }_{o}$$

where K is the cell constant which is a function of cell dimensions, (*ε*_o_) is the permittivity of free space (6). The difference between the values ε′_s_ and ε′_∞_ at low and high frequencies is called “The dielectric increment Δε′, providing a measure for the shape and volume of the non polar solution (11).

The dielectric loss ε″ was calculated from the relation:

(i) ε//=ε//tanδ and tanδ=1/2πfRC

The conductivity σ is calculated by the equation:

(j) $$\sigma =K/R\left(\Omega^{-1}m^{-1}\right)$$

where R is the resistance of the given sample. This implies that permittivity and conductivity can not vary independently with frequency. The conductivity σ usually has a frequency – independent part (due to ionic conduction) plus a frequency – dependent part (due to dielectric relaxation).

Relaxation time (τ) can be calculated from the relation:

(k) $$\tau =1/2{\pi f}_{c}$$

where *f_c_* is the critical value of the frequency that corresponds to the mid point of the dispersion curve. The relaxation time is proportional to the square of the molecule radius:

(l) $$\tau =e_{o}r^{2}/2\mu \mathrm{kT}$$

where μ is the surface mobility of the counter ions (m^2^/V sec), e_o_ is the charge of the counter ion, k is Boltzman constant and T is the temperature of the sample in Kelvin. When an electric field is applied, the ions in the system will redistribute under the influence of the field and the diffusion (8, 9). Gross (8, 9) obtained a set of coupled differential equations for the ion concentrations and current densities. The solution of these equations yields broad, asymmetrical, low frequency dispersion in the permittivity of the particle.

The time constant τ of this dispersion is similar to that derived by Schwarz (19).

(m) $$\tau =r^{2}/D$$

where D is the diffusion coefficient of ions. The radius (r) of the molecule is given by:

(n) $$r^{3}=\mathrm{KT}\tau /4\pi \eta$$

η is the viscosity of the sample. The dielectric relation in the frequency range of KHz and below was known by the ∝- dispersion (7), is mainly due to counter ions molecules of hemoglobin.

**Material and Methods.** Forty two male Albino rats in the weight range of 100-120 g maintained on standard laboratory diet were used. Rats were randomized into 6 groups (7 rats/group). The following groups were studied; (a) normal rats; (b) rats exposed to 0.3 mT for one week; (c) rats exposed to 0.5 mT for one week; (d) rats exposed to 1 mT EMF for one week.

Blood samples were directly collected from the eye - vein of rats using heparinated capillary tubes. The heparinized blood was centrifuged at 3500 r.p.m. for 10 minutes at 4°C, plasma was harvested and stored at 20°C for further investigation. The packed red blood cells (RBCs) were hemolyzed to produce hemoglobin (Hb) following the method cited by Trivelli *et al* (22).

**Figure 1:**
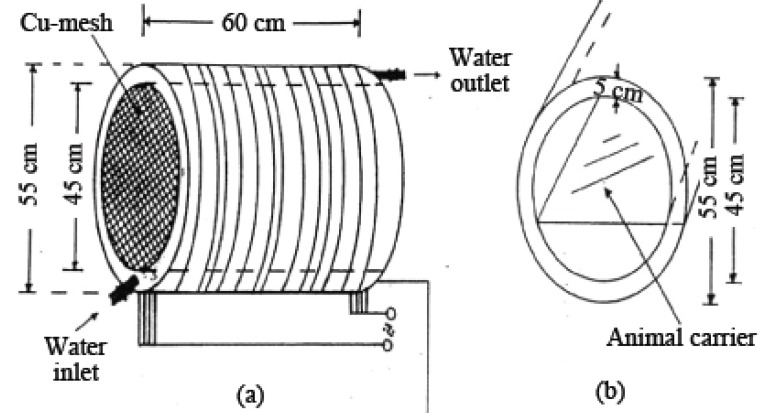
A diagram of the magnetic exposed system. Animals were exposed continuously as a group in plastic cage on the shelf within the solenoid.

**Exposure facility.** Animals were exposed to a homogenous magnetic field generated by 16 coils. Coils were composed of 115 turns of Cu wire of 1.2 mm diameter. Water was pumped in a copper jacket separating the wire winding and the chamber in order to maintain constant temperature (Figure 1). There was no measurable difference in temperature between the room and the chamber (within ± 0.1°C). The animals were maintained in special plastic cages that permit normal ventilation and daylight, and cages were fixed on supports inside the irradiation chamber. Food and water were kept in special open containers fixed to the walls of the cages. Animals of group A were housed in a similar cage, kept during the run of the experiment in a typical cylindrical chamber made of copper having the same dimensions of the exposure facility, permitting exactly similar illumination and ventilation for both control and exposed groups. The chamber for control group was well grounded.

The magnetic field exposure system was locally manufactured (Cairo University). The magnetic field intensity was measured by means of a hand held Gauss/Tesla meter with accuracy of 0.2% (model 4048 with prop T-4048 manufactured by (W. Bell) in U.S.A.

**Osmotic Fragility**. Osmotic fragility test of whole blood was performed for control and experimental groups according to Dacie and Lewis (3). Tests were carried out within two hours of sample collection. Whole blood was added to varying concentrations of buffered sodium chloride solution NaCl buffered to pH7.4 and kept at 25°C. The amount of hemolysis was then determined by reading the supernatants absorbance by means of spectrophotometer (UV/ visible spectrophotometer Jasco V-530, made in Japan) (2).

**Viscosity Measurements.** The Brookfield DV-III Programmable Rheometer (cone and plate) measures blood parameters of shear stress and viscosity in centipoise (cP) under controlled conditions of shear rates, temperature and time. The sample volume required for this experiment was 0.5 ml. of fresh heparinized blood. Computer controlled measurement protocols allow for ease of operation and reproducible measurement conditions. The shear rate of a given measurement is determined by: the rotational speed of the spindle, the size and shape of the spindle, the size and shape of the container used, and therefore, the distance between the container wall and the spindle surface.

## RESULTS AND DISCUSSION

### Influence of EMF on Viscosity

**Figure 2:**
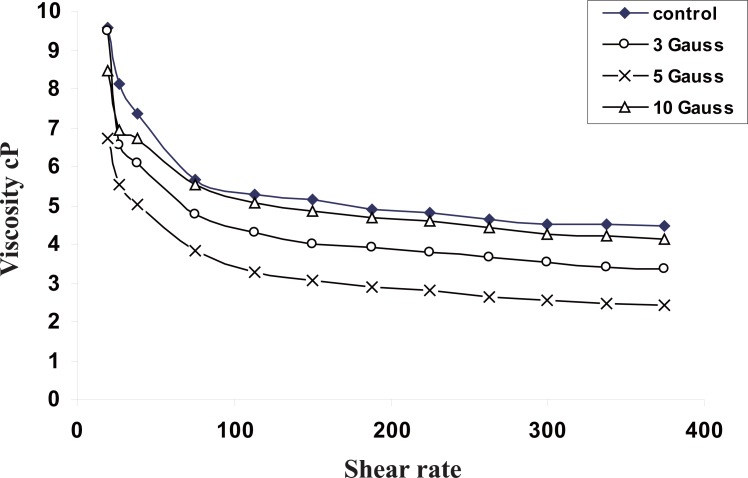
Shear rate dependence of viscosity of normal blood and exposed animals to different electromagnetic fields (3,5 and 10 gauss).

Figure 2 illustrates the shear rate dependence of the blood viscosity in (cP) of normal blood and exposed samples to different electromagnetic field doses (3, 5 and 10 mT).

Thurston (21) showed that hematocrit HCT is a strong parameter for establishing blood viscosity because of its central role of the elastic red cell, and normally there is a proportional relation between HCT and viscosity (the increase in HCT means increase in RBCs). Table 1 illustrates the hematological data from rats (control), exposed to 0.3, 0.5 and 1 mT electromagnetic 50 Hz. RBCs: Red blood cells. MCV: Mean Corpuscular Volume. MCH: Mean Corpuscular Hemoglobin. MCHC: Mean Corpusclar Hemoglobin Concentration. HCT%: Hematocrite Percentage. Hb: Hemoglobin. TLC: total white blood cells. The results indicate that for rats exposed to EMF, a significant change was observed in total red blood cells, hemoglobin levels, and hematocrite values. There was also remarkable increase in TLC with the increase of electromagnetic field value.

**Table 1:** Illustrate the hematological data from rats (control), exposed to 0.3 ,0.5 and 1 mT electromagnetic 50 Hz

	Control	Exposed to 3 Gauss (0.3 mT)	Exposed to 5 Gauss (0.5 mT)	Exposed to 10 Gauss (1 mT)

***Hb (g/dl)***	12. 2 ± 0.06	11.65 ± 0. 06	11.1 ± 0.03	11.5 ± 0.02
***RBCs (melion/ul)***	4.4 ± 0.2	4.7 ± 0.05	5.6 ± 0.02	4.5 ± 0.02
***TLC (X100/ul)***	9.2 ± 0.9	9.9 ± 0.9	13.5 ± 0.05	19.25 ± 0.3
***HCT %***	37.2 ± 1.1	41.2 ± 0.09	46.50 ± 0.05	37.9 ± 0.025
***MCV (fl)***	83.3 ± 0.48	87.04 ± 0.004	83.0 ± 0.025	84.2 ± 0.055
***MCH (PG)***	27.7 ± 0.13	29.01 ± 0.04	27.6 ± 0.07	28.1 ± 0.3
***MCHC (g/dl)***	33.3 ± 0.03	33.3 ± 0.025	33.3 ± 0.03	33.3 ± 0.04

**Figure 3:**
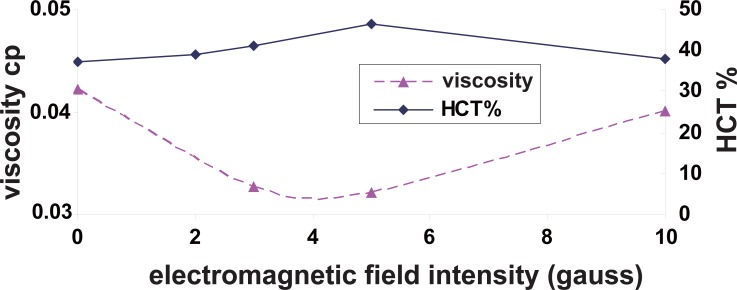
The variation of blood viscosity and HCT% due to electromagnetic exposure.

To examine how viscosity changes with hematocrite in the presence of electromagnetic field, values of viscosity and hamatocrite were plotted versus the electromagnetic field (Figure 3). It was observed that the viscosity decreased as the hematocrite HCT% increased under the effect of electromagnetic fields.

### Influence of EMF on red cell deformability

**Figure 4:**
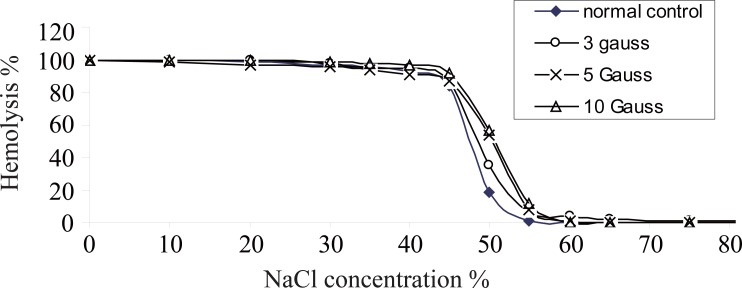
The variation of the percentage hemolysis for the RBCs as a funcio of the NaCl concentration % for samples collected from animals (control, exposed to different EMF doses.

**Figure 5:**
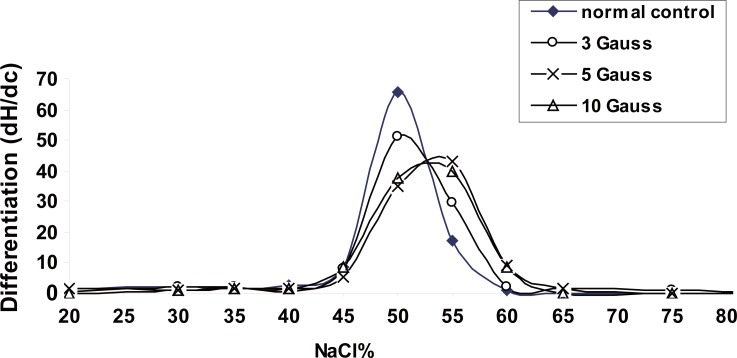
Differentiation curves for Figure 4.

In order to study the effects of electromagnetic field exposure on the cell rigidity, red cells can be modified by hardening and by changing osmotic pressure (Figure 4), which shows the effects of electromagnetic field on the osmotic fragility measurements of RBCs collected from animals of the different groups. By the addition of hypotonic saline to swell the cells, the percentage of hemolysed cells is plotted as a function of the concentration percentage of NaCl. For analysis of these results, the curves were differentiated and plotted as a function of NaCl concentration percentage as shown in Figure 5. As a result of this treatment each characteristic plot was represented by a peak whose width indicates the elastic range of the RBCs cellular membrane.

The percentage of NaCl concentration C% at which hemolysis starts to occur characterizes the transport of water molecule through the RBCs membrane and hence its permeability. The widths at half maximum W_hmax_ of these differential plots represent the relative elastic limit of RBCs membrane. The increase of W_hmax_ will represent the increase of cellular membrane elasticity. The average value of C% and W_hmax_ for RBCs from each group is given in Table 2. The data indicates that the permeability of RBCs increased only with the exposure to 5 gauss while at this dose the cellular membrane elasticity decreased as previously demonstrated by Fadel *et al* (4). The viscosity of blood depends mainly on the membrane elasticity of RBCs. Therefore, decrease of RBCs membrane elasticity will lead to the increase of the blood viscosity. Also the remarkable decrease in the elasticity of the RBCs membrane may lead to increased resistance of capillaries to the passage of RBCs, potentially promoting toxic effects on some organs.

**Table 2:** The average value of C% and W_h max_ for RBCs from each group are given (male rats)

Group	*C%*	*W_h max_*

Normal control	55%	0.15
Exposed to 3 Gauss (0.3 mT)	55%	0.15
Exposed to 5 Gauss (0.5 mT)	60%	0.10
Exposed to 10 Gauss (1 mT)	55%	0.11

### Influence of EMF on the molecular structure of Hb

**Figure 6:**
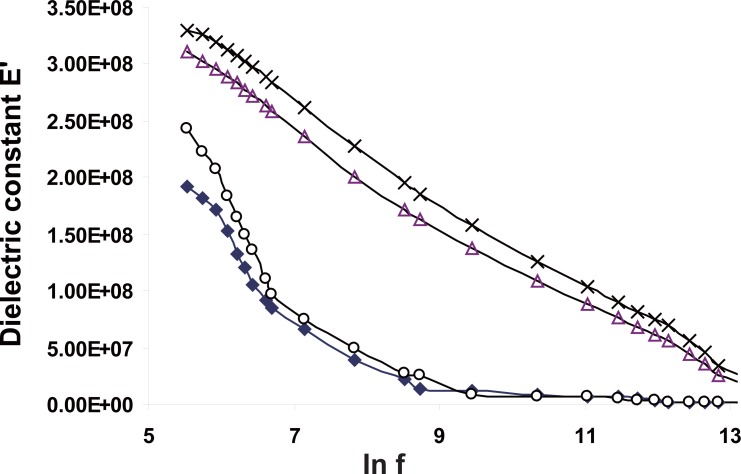
The variation of the dielectric constant E' for Hb from all groups as a function of the applied frequency.

The variations of dielectric constant ε′ as a function of frequency in range 50 Hz up to 5 MHz are shown in Figure 6. It is clear that ε′ decreases monotonically with increase in frequency for all groups. This behavior can be described by the Debye dispersion relation,

$\varepsilon^{/}=\varepsilon_{\infty }^{/}+\left\{\left(\varepsilon_{s}^{/}-\varepsilon_{\infty }^{/}\right)/\left(1+\omega^{2}\tau^{2}\right)\right\}$

The behavior of ε′ can be explained as a normal behavior obeying the Debye model in which the dipoles can easily switch alignment with changing fields at low frequency. As the frequency increases the dipoles are less able to rotate and maintain phase with the field. Thus they reduce their contribution to the polarization field, and hence the observed reduction in the real part ε′. It can be noted that the changes in the value of $\varepsilon_{s}^{/}-\varepsilon_{\infty }^{/}$ are functions of the changes in the dipole moment of the Hb molecules which will consequently depend on the centre of the mass of the charge distribution of the electric dipole (11).

**Figure 7:**
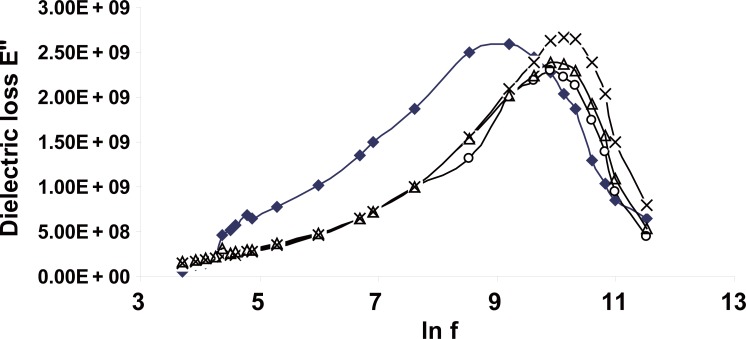
The variation of the dielectric loss E″ of Hb for all groups as a function of frequency.

Figure 7 shows the variation of the dielectric loss ε″ of all investigated samples versus frequency, 50 Hz up to 5 MHz. It should be noted that the dielectric loss ε″described the Debye dispersion relation,

$\varepsilon^{"}=\left\{\left(\varepsilon_{s}^{/}-\varepsilon_{\infty }^{/}\right)\left(\omega \tau \right)\right\}/\left(1+\omega^{2}\tau^{2}\right)$

where ε″ rises with the increase of frequency and at the critical frequency f_c_ the losses get to reduce because the field frequency begins to exceed their characteristic natural frequency. It is clear from the figure that this critical frequency f_c_ (the middle point of the dispersion curve) was changed from the control for each exposure dose and then resulted in changes in the relaxation time τ of the samples.

**Figure 8:**
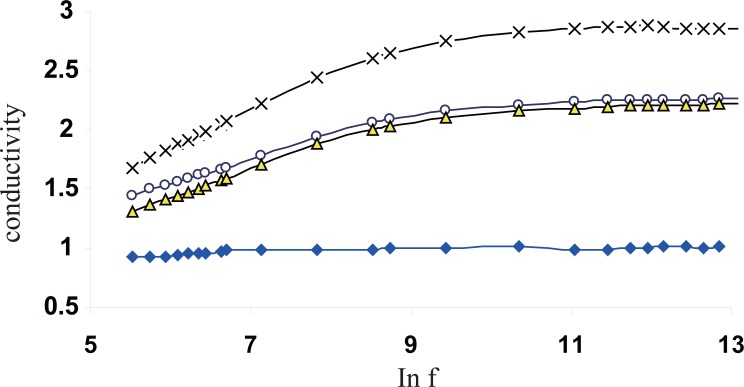
The variation of the conductivity of Hb for all groups as a function of frequency.

Figure 8 shows the variation of the total electrical conductivity σ with the applied frequency in the range of 50Hz up to 5 MHz, for all groups. It is clear from the figures that the decrease in the value of the ε′ was accompanied by an increase in the value of the conductivity (7).

For all samples, an increase of conductivity values for all exposed groups specially the dose of 5 gauss could be observed. It is clear that the electric conductivity of the RBCs of the exposed groups is larger than the control.

The relaxation time τ (msec), the radius of Hb molecule r (nm), the conductivity σ, diffusion coefficient of ions D and the surface mobility μ were calculated for all groups are given in Table 3.

**Table 3:** The average values of the relaxation time (τ), radius (r), conductivity (s), charge distribution (D) and the surface mobility μ for different groups

Group	τ (μsec)	R (nm)	σ (s/m)	d = τ/r^2^	U (m^2^/vsec)

Normal control	0.79 ± 0.2	6.39 ± 0.003	0.18 ± 0.006	0.24 ± 0.05	79.08 ± 0.02
Exposed to 3 Gauss (0.3 mT)	1.59 ± 0.01	13.5 ± 0.015	0.23 ± 0.07	0.19 ± 0.05	99.7 ± 0.09
Exposed to 5 Gauss (0.5 mT)	0.531 ± 0.02	5.58 ± 0.015	0.28 ± 0.005	0.17 ± 0.01	114.06 ± 0.09
Exposed to 10 Gauss (1 mT)	0.636 ± 0.05	5.9 ± 0.05	0.221 ± 0.01	0.18 ± 0.03	106.4 ± 0.07

The data indicate that exposure of animals to the magnetic fields caused structural changes in Hb, which may affect their properties and hence RBCs physiological functions.

The results indicate a strong dispersion in the β region for all the samples from all groups studied. The dielectric dispersion in the β range (0.1-5 MHz) is mainly due to protein and counter ion molecular relaxation. Moreover the results indicted that the values of the r, τ, σ and μ for Hb molecule from exposed group to the magnetic field (3 gauss) were higher than control while the relaxation time and the radius of the Hb molecule decreased than that of the control for the group exposed to 5 and 10 gauss. This provides a may explain why the viscosity at 5 gauss was higher than that at 3 gauss. One may conclude that the dose 3 gauss may cause more damage than the 5 and 10 gauss exposures which may be used in such treatment.

Since RBCs membrane play an essential role in the blood flow rate, changes in its biophysical properties will affect its capability for carrying on its metabolic functions since the RBCs counts were unchanged after exposure of the animals to the magnetic field.

## CONCLUSION

In the present work, exposure to EMFs is found to produce conformational changes in hemoglobin.

DNA oscillates in the normal state of the cell. Thus, we may expect that electromagnetic fields may also affect its oscillation which may also reduce the activation of RNA which responsible to the production of protein. Such reduction in the protein molecules decreased the blood viscosity where the blood flow rate increased. This decrease of such proteins is responsible of the RBCs membrane hardness and its deformability. EMFs may also activate bone marrow cells to produce more RBCs. It is known that as the number of RBCs increase, blood viscosity also increased although the RBCs increased the viscosity decreased .This may be due to activation of the Rouleau formation of RBCs which depend on the electric charges and responsible of viscosity decreasing. This may be a key for further research in the area of the effect of EMF on the plasma proteins.
